# Tubo-ovarian abscess in a virgin girl

**Published:** 2011

**Authors:** Tahere Ashrafganjooei, Iraj Harirchi, Giti Iravanlo

**Affiliations:** 1 Department of Obstetrics and Gynecology, Afzali-poor Hospital, Kerman University of Medical Sciences, Kerman, Iran.; 2 Department of Surgical Oncology, Tehran University of Medical Sciences, Tehran, Iran.; 3 Department of Clinical Pathology, Tehran University of Medical Sciences, Tehran, Iran.

**Keywords:** *Tubo*-*ovarian abscess*, *Virgin*, *Vaginal septum*

## Abstract

**Background:**

Tubo-ovarian abscess as a serious complication of pelvic inflammatory disease is very uncommon in sexually inactive girls.

**Case:**

We report a case of tubo-ovarian abscess in a 24-year-old sexually inactive girl with transverse vaginal septum who was presented with abdominal pain and a pelvic mass and without prior surgical history and no evidences of appendicitis, inflammatory bowel disease, or cancer. A huge unilateral tubo-ovarian abscess was recognized at laparotomy. Unilateral salpingoophorectomy, hysterectomy and postoperative antibiotic therapy cured the patient.

**Conclusion:**

Early diagnosis and treatment are essential to prevent further sequel including infertility, ectopic pregnancy, and chronic pelvic pain which cause morbidity.

## Introduction

Pelvic inflammatory disease (PID) is a complex polymicrobial infection. It is thought to arise from ascending of microorganisms from the lower genital tract to the endometrium and fallopian tubes, pelvic peritoneum and adjacent structures, causing inflammation (1). It is generally a disease of young, sexually active women of reproductive age group (2). It has traditionally been thought that sexual activity is a prerequisite for acquiring this disease thus, PID is considered to be rare in sexually inactive girls (3). Our review of the literatures revealed 10 cases of tubo - ovarian abscesses in virgins (3-8).

Tubo-ovarian abscess (TOA) is an end-stage process and a serious manifestation of PID that usually reflect an agglutination of pelvic organs (tube, ovary, bowel) forming a palpable complex (9). They may also occur after pelvic or abdominal surgery by spreading of organisms from adjacent gastrointestinal infections or occasionally form super infection of a necrotic ovarian malignancy, so; postmenopausal women presenting with TOAs, should be thoroughly investigated to exclude a concomitant pelvic malignancy and conservative treatment of TOAs has no place during the menopause (10). The correct diagnosis of TOA is usually made during laparotomy and patient history. Physical examination and diagnostic tests are relatively non-specific (2). 

Treatment of TOA involves the use of an antibiotic regimen administered in a hospital. About 75% of women with TOA respond to antimicrobial therapy alone. Failure of medical therapy suggests the need for drainage of the abscess (1). Laparoscopic surgery which minimizes postoperative complications can be the first option in the treatment of TOA (5). Percutaneous drainage guided by imaging studies (ultrasound or computed tomography scan) has also been used successfully (1).

## Case report

A 24 year- old blind virgin, with history of mental retardation, primary amenorrhea and normal secondary sexual development, was presented to infectious ward of Imam Khomaini Hospital, Tehran, Iran; with severe right lower quadrant pain, diarrhea and fever since two days ago. 

Her mother mentioned a history of lower abdominal pain, low-grade fever, and medical therapy in her since a few months ago. In physical examination, external genitalia were grossly normal and hymen was intact, but rectal examination revealed blind vagina of about 1 cm length and an ill-defined right pelvic mass. Abdominal palpation revealed moderately tenderness in lower abdomen particularly in right lower quadrant.

Laboratory analysis showed a white blood cell count of 188000/µl, hemoglobin level of 11.5g/dl, ESR=121 and negative blood and stool culture. The chest radiograph was negative. Preoperative transabdominal sonogram and computed tomographic scan of the abdomen and pelvis were done. 

**Figure 1:**
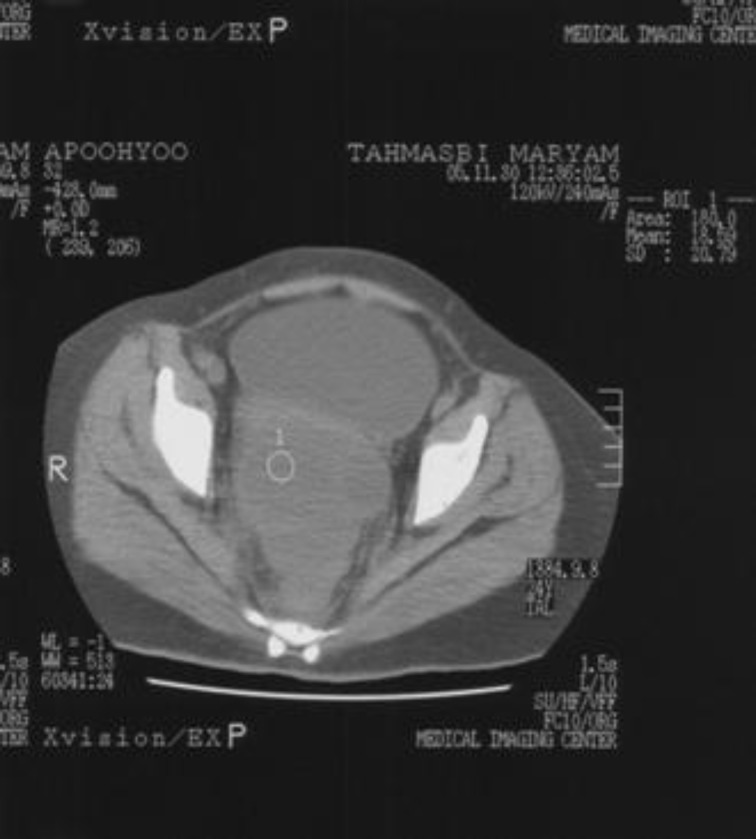
Preoperative CT-Scan demonstrating an ill-defined pelvic mass

The sonogram demonstrated a 7×7.5 cm complex mass in right pelvis with irregular borders and multiple septa. Dilation of the right urinary collecting system due to extrinsic compression was noted on sonograghy. Abdomino pelvic CT-Scan confirmed ultra sonographic findings (Figure 1).

The patient was placed on intravenous ceftriaxone, but subsequently developed spiking fever after 5 days. On abdominal exam, she developed severe tenderness and rebound. We discussed with her family and emphasized the clinical suspicion of pelvic tumor necrosis and abscess formation or to lesser degree, apandicular abscess and the need for surgical resection.

She underwent exploratory laparotomy. The abdomen was free of frank pus and adhesions, but there were severe adhesions in pelvis. After release of adhesions, about 300 cc greenish yellow, pus like foul smelling material was coming from right adnexal swelling into the pelvic cavity. Pus was drained and exploration revealed a10×12cm right-sided pelvic mass with extensive adhesions to the recto-sigmoid. 

The mass was identified as a right tubo-ovarian abscess, with no evidence of diverticular or appendiceal diseases at surgical inspection. All abdominal organs were found to be normal. Right salpingo-oophorectomy, was performed. During surgery pus was removed from TOA and transported to the laboratory, cultured for both anaerobic and aerobic organisms. Due to her mental retardation, after consent of her mother, the patient underwent total abdominal hysterectomy and then peritoneal lavage was done. The thin lower third vaginal septum also was resected. The culture of pus was positive and mixed. 

Postoperatively, the patient received ceftriaxone 1g i.v. every 8 hr plus clindamycin 900 mg i.v. every 8 hr. She defervesced gradually and became afebrile after three days. Parenteral antibiotics were replaced with oral antibiotics 72 hours later and recommended for 14 days. Histophatologic analysis showed a right tubo-ovarian abscess with severe inflammatory changes and necrosis with marked neutrophil infiltration. Postoperative recovery was uneventful and the patient was discharged on the fifth postoperative day.

## Discussion

Tubo-ovarian abscess is an advanced form of pelvic inflammatory disease, most often caused by spread of bacteria from the lower genital tract (9). TOA is a serious complication of pelvic inflammatory disease rarely seen in sexually inactive girls and it is generally the result of a blood-borne or gastero-intestinal infection such as appendicitis in these cases (5). It can also develop as a complication of pelvic or abdominal surgery and malignancy (8). 

The infection in TOA is usually polymicrobial with mixed aerobic-anaerobic organisms. The clinical presentation of TOA tends to be non-specific. Vaginal discharge is present in less than 5% of patients. The typical ultra sonographic appearance of a tubo-ovarian abscess is a multilocular, cystic and complex adnexal mass often with debris and thick septations. Hartmann reported two cases with purulent fluid collections in the fallopian tubes that were not evident on imaging, but the laparoscopic evaluation revealed them (7). 

Therefore he recommends the use of laparoscopy for diagnosis in atypical cases. Ruptured TOA is associated with a high risk of development of septic shock, in case that urgent surgical intervention is not undertaken (6). Broad spectrum antibiotics should be given promptly to cover polymicrobial mixed aerobic/anaerobic infections. Prompt surgical treatment may be associated with decreased morbidity and postoperative hospitalization. Extensive inflammation and edema makes the dissection of pelvic abscess difficult. 

Complications of tubo-ovarian abscesses include tubal occlusion, increasing the risk of infertility and ectopic pregnancy, on the other hand, pelvic adhesions leading to chronic pelvic pain. Early diagnosis and surgical treatment are essential to prevent further sequela and improving outcome.

We must consider TOA in the differential diagnosis for abdominal pain and pelvic mass with fever in adolescent females regardless of sexual history.

## References

[1] Soper DF, Berek JS (2007). Genitourinary infections and sexually transmitted diseases. Novak's Gynecology.

[2] Beigi RH, Wiesenland HC (2003). Pelvic inflammatory disease: new diagnostic criteria and treatment. Obstet Gynecol Clin North Am.

[3] Hartmann KA, Lerand SJ, Jay MS (2007). Tubo-Ovarian Abscess in Virginal Adolescents: Look for Underlying Etiology. J Pediatr Adolesc Gynecol.

[4] Dogan E, Altunyurt S, Altındag T, Onvural A ( 2004). Tubo-ovarian Abscess Mimicking Ovarian Tumor in a Sexually Inactive Girl. J Pediatr Adolesc Gynecol.

[5] Arda IS, Ergeneli M, Coskun M, Hicsonmez A (2004). Tubo-ovarian abscess in a sexually inactive adolescent patient. Eur J Pediatr Surg.

[6] Teng FY, Cardone JT, Au AH (1996). Pasteurella Multocida tubo-ovarian abscess in a virgin. Am J Obstet Gynecol.

[7] Hartmann KA, Lerand SJ, Jay MS (2009). Tubo-ovarian abscess in virginal adolescents: exposure of the underlying etiology. J Pediatr Adolesc Gynecol.

[8] Gensheimer WG, Reddy SY, Mulconry M, Greves C (2010). Abiotrophia/Granulicatella tubo-ovarian abscess in an adolescent virginal female. J Pediatr Adolesc Gynecol.

[9] McNeeley SG, Hendrix SL, Mazzoni MM, Kmak DC, Ransom SB (1998). Medically sound, cost-effective treatment for pelvic inflammatory disease and tuboovarian abscess. Am J Obstet Gynecol.

[10] Khan NA, Maajeeni EH (2005). Tubo-ovarian abscess in a postmenopausal woman with underlying ovarian carcinoma. Saudi Med J.

